# Perceptions of the Government’s Response to the Pandemic: Voices From the COVID-19 Coping Study

**DOI:** 10.1093/geroni/igab046.567

**Published:** 2021-12-17

**Authors:** Haley Gallo, Lindsay Kobayashi, Jessica Finlay

**Affiliations:** 1 USC, Discovery Bay, California, United States; 2 University of Michigan, Ann Arbor, Michigan, United States

## Abstract

The COVID-19 pandemic was met with conflicting government strategies in the handling of the virus. Older adults were disproportionately impacted by the pandemic, yet little is known about their perspectives of the government response. Using data collected in September and October, 2020 from the online, nation-wide COVID-19 Coping Study, we conducted qualitative thematic analysis on a subsample of respondents (N=500) proportionate to the age, gender, race/ethnicity, and education of the U.S. population age 55+. Two researchers individually coded a random subsample of 50 open-ended responses to the question “How do you feel about federal government responses to and handling of the COVID-19 pandemic?” Using NVivo qualitative software, the researchers compared codes and reconciled differences to achieve a Kappa score of >0.8. The first author coded the remaining responses using the established coding strategy. Analyses identified themes related to President Trump’s leadership, Congress, the broader federal government, and science. Some participants indicated that the federal government’s response to the pandemic was “inadequate,” “too political,” and “lacking coordination.” Others expressed that the president “did the best he could” or that “it’s not the federal government’s responsibility.” While some praised vaccine development efforts and expressed their appreciation for Dr. Fauci, others expressed scientific distrust. Participants’ perspectives were divergent, reflective of the country’s polarization surrounding COVID-19 policies and practices. Differences in perspectives exist by race/ethnicity, gender, geographic region, and age. Study results can help identify groups of older adults who may need targeted programs and policy support.

